# Gastrotomy

**Published:** 1863-08

**Authors:** 


Gastrotomy. Large abdominal uterine tumor extirpated by John O'Reilly, M.D., F.R.C.S.I.

This is a brief but interesting report of a case of "fatty-fibro-cellular" tumor, weighing over *thirty* pounds, originating in the lower part of the abdomen. The patient lived nine days after the extirpation.

